# The Microscope: And Its Relations

**Published:** 1856-11

**Authors:** 


ART. III.— *The Microscope: and its Relations.* By Wm. B. Carpenter, M. D, F. R. S., F. G. S., etc., etc., etc. With an Appendix containing the Applications of the Microscope to Clinical Medicine, etc. By Francis Gurney Smith, M. D., Professor of the Institutes of Medicine in the Medical Department of Pennsylvania College, etc. Illustrated by 100 Engravings on Wood Philadelphia: Blanchard & Lea. 1856.

Very much of the perfection of any science depends upon its working-tools. The triumphs of modern engineering, that vast system of triangulation which has mapped out so accurately the dangers of our coasts, and the most valuable of astronomical discoveries, are all attributable more or less to the exactitude with which scientific instruments are constructed, and most of them would have been impossible without the aid of such instruments. Without the telescope, who would have solved the problem of the nebulæ or discovered Neptune? But what the telescope has done in overcoming distance and magnitude, its reversed self, the microscope, has accomplished with the opposite difficulties of proximity and minuteness. If the one has opened the path through space to new firmaments, and drawn the veil which covers distant worlds, the other has given us a more exceeding astonishment in the worlds within worlds, the life within life, the endless lengthening and multiplication of the links of that unbroken chain which reaches from dust to Deity, which it reveals. Our author has chosen an intelligent and meaning word when he speaks of the *revelations* of the microscope. It is truly a revelation—an evangel of knowledge.

We have little patience with those philosophers who reject the microscope as a means of diagnosis in disease. So soon as the first furor subsided, so soon as men learned that the microscope was not in itself the All-Seeing-Eye, they grew impatient and cast aside the idol they had worshipped. It is a daily occurrence, now, to read and hear remarks deprecatory of its value in disease, for reasons which apply equally to all our powers of investigation.

It is imperfect. So is everything but the Supreme Architect. It is contradictory. So are the indications of the pulse. It never tells the whole truth. Neither does physical diagnosis. It stops short—leaves mysteries unsolved as deep as those it teaches. How much more true is this of the human eye. Our powers of vision are multiplied a thousand fold, and yet we are willing to cast away our new endowment, because a thousand-fold is not the measure or the depth of the problem.

We are willing to be content with the microscope. It has its fallibilities, its weaknesses; it is rarely competent to the diagnosis of disease when unassisted, but it is a most useful assistant; it can settle many questions of grave importance which must otherwise remain unanswered. We do not admire the spirit of those practitioners who reject it because it falls short of perfection. Neither will we willingly rank as one of its blind admirers, who are willing to stake a life on the results which it affords. The old quotation—the middle path is safest—is true forever.

But this is not a review of Dr. Carpenter's book. Of it, we may say that Dr. Carpenter is one of those best qualified to write upon this subject. His studies as a physiologist have taught him the use and value of the instrument. He criticizes its merits as a tool, and as might have been expected, gives the palm to the English instrument. In everything except the element of cheapness, it does, indeed, rank high; though we are at a loss to conceive why it should be preferred to many others. Those of Nachet are cheap, accurately mounted, and, as a general thing, are good definers. Those of American make, the Spencer instruments, are unrivalled for achromatic quality, and power of definition, though miserably deficient in accessories and mounting. Those made by Powell and Lealand, the only English instrument with which we are familiar, have combined merits. In the low powers we consider them a better working instrument than any other. The Dissecting Microscope, by Powell and Lealand, is unrivalled in this respect. For use in anatomical labor, it is so convenient, so little time is required for adjustment, that it has become our especial favorite.

Dr. Carpenter has evidently intended this rather as a working manual, than as a special treatise for the medical man. In his classification of the work we find that he considers in order the history of the microscope and micrograpic research, the optical principles of the microscope, its construction, its accessory apparatus, its management, and the preparation, mounting and collection of objects.

Taking up next its application to scientific purposes, he gives the microscopic forms of vegetable life, the protophytes, the higher cryptogamia, and the phanerogamic plants. Turning to animal life he begins with the protozoa and animalcules, the foramenifera and sponges, the zoophytes, the edimodermata, the polyzoa, mollusca, worms, crustacea, insects, vertebrated animals, and closes with two chapters devoted to the geological applications of the microscope, and its uses in the inorganic or mineral kingdom.

It will be seen in this enumeration, that no attention has been given to the microscope in disease. This hiatus is very ably filled by Prof. Smith, the American editor, who gives an appendix affording a concise and valuable summary of its use in diagnosis. The addition is valuable, and will commend the book to many who would otherwise have regarded it rather as a source of interest, than as an intrinsic addition to their means of usefulness.

With a word of praise for the publishers, who have issued this work in so neat and elegant a form, and have so copiously illustrated its pages, we commend it to our readers as a book worth purchasing.

For sale by PHINNEY & Co.

